# The Effect of JAK Inhibitors on Patient-Reported Outcomes in Psoriatic Arthritis

**DOI:** 10.31138/mjr.171223.tej

**Published:** 2024-03-30

**Authors:** Sotirios G. Tsiogkas, Carlo Perricone, Dimitrios P. Bogdanos

**Affiliations:** 1 Department of Rheumatology and Clinical Immunology, Faculty of Medicine, University of Thessaly, University General Hospital of Larissa, Greece,; 2 Section of Rheumatology, Department of Medicine and Surgery, University of Perugia, Italy

**Keywords:** patient-reported outcomes, tyrosine kinase 2, Janus kinase inhibitors, JAK, psoriatic arthritis

## Abstract

**Objective:**

Psoriatic arthritis (PsA) is a chronic inflammatory disease that affects the joints and skin of patients with psoriasis. In this review we aimed to summarise the available evidence regarding the effect of Janus kinase inhibitors (JAKi) on patient-reported outcomes (PROs) when used for the management of PsA.

**Methods:**

We utilised a narrative review approach as we searched the available literature for articles to be included in our study.

**Results:**

JAKi have been found to be effective in inducing better PRO responses compared to placebo. These findings have been consistent across various patient populations, including those with active PsA, those with an inadequate response to conventional therapies, and those with comorbidities. The evidence supporting the benefits of JAKi on PROs in PsA is compelling, demonstrating consistent improvements in pain, physical function, fatigue, and quality of life.

**Conclusion:**

Numerous studies have demonstrated the the efficacy of JAKi in improving PROs in patients with PsA.

## INTRODUCTION

Psoriatic arthritis (PsA) is a chronic inflammatory disease that affects the joints and skin of patients with psoriasis.^1^ The exact prevalence of PsA is unknown, but estimates vary from 0.3% to 1% of the population.^2^ PsA often develops within 10 years of developing psoriasis, a disease characterised by hyperkeratotic plaques on the skin.^3^ Psoriasis affects approximately 2% of the population and up to 30% of them may develop PsA over time. The disease usually affects larger joints, especially those of the lower extremities and the distal joints of the fingers and toes.

PsA is a progressive and potentially disabling disease that can impair the quality of life of patients.^4^ Without any treatment, PsA can lead to permanent joint damage and deformity. Therefore, early diagnosis and treatment are essential to control the symptoms and prevent further complications.

The aetiology and pathophysiology of PsA are not fully understood, but they are thought to involve a complex interplay of genetic and environmental factors that trigger an abnormal immune and inflammatory response.^5^ Several genes have been associated with PsA susceptibility and severity, such as HLA-B27, IL23R.^6^ Environmental factors that may influence PsA development and progression include infections, trauma, stress, smoking, and alcohol consumption.^7^ The molecular mechanisms of PsA involve the activation of various cytokines and signalling pathways that mediate joint inflammation, synovial hyperplasia, bone erosion, cartilage degradation, and skin lesions.^8^

Janus kinase inhibitors (JAKi) are a novel class of therapeutics that exert their immunomodulatory effects by targeting the Janus kinase (JAK)/signal transducer and activator of transcription (STAT) signalling pathway.^9^ This pathway is essential for cytokine signalling, which plays a pivotal role in the pathogenesis of a wide range of auto-immune and inflammatory diseases, including PsA. JAKi act by competitively binding to the ATP-binding site of JAK enzymes, thereby preventing their phosphorylation and activation. This inhibits the phosphorylation and translocation of STATs to the nucleus, where they act as transcription factors to regulate the expression of genes involved in inflammation, immune cell development, and cell growth. By inhibiting JAK enzymes, JAKi block cytokine signalling and reduce the production of pro-inflammatory mediators. Different JAKi have different selectivity for the four members of the JAK family (JAK1, JAK2, JAK3, and TYK2), which are associated with different cytokine receptors and immune cell subtypes. Collectively, those immunomodulatory effects of JAKi contribute to their therapeutic efficacy in PsA. The therapeutical group has recently been shown to significantly improve clinical outcomes in PsA patients, including reduction of joint pain and swelling, improvement of physical function, and saving the need for other immunosuppressive medications.^11–13^

Since PsA has a major impact on quality of life of patients, patient-reported outcomes (PROs) are important components in the assessment of therapeutic efficacy for PsA. In this review we aimed to summarize the available evidence regarding the effect of JAKi on PROs when used for the management of PsA. In that context, we also provide an overview of the currently investigated effect of JAKi on core PRO markers such as pain and depression in translational research.

## METHODS

We utilised a narrative review approach as we searched the available literature for articles to be included in our study. We searched MedLine (through PubMed) until November 2023. We included articles that we judged as relevant to our research question based on our clinical experience, a process possibly prone to selection bias (**Table 1**).

**Table 1.** Study characteristics.

**Study**	**Origin**	**Allocation**	**Outcome assessment timepoint (wks)‡**	**Study Arms**	**Reported outcomes‡**	**Randomised**	**Sponsor**
EQUATOR 2018^23^	Multicentre	1:1	16	1. filgotinib 200 mg once daily 2. placebo	PsAID9; SF-36 PCS; SF-36 MCS	131	Galapagos NV
Leng 2023^20^	China	2:1	12	1. tofacitinib 5 mg twice daily 2. placebo	PtGA-VAS; Pain-VAS; HAQ-DI; SF-36 PCS; SF-36 MCS	204	Pfizer
Mease 2021^24^	Multicentre	1:1	16	1. deucravacitinib 6 mg once daily 2. deucravacitinib 12 mg once daily 3. placebo	Pain-VAS; PtGA-VAS; HAQ-DI; SF-36 PCS; SF-36 MCS	203	Bristol-Myers Squibb
OPAL Beyond 2017^18^	Multicentre	2:2:1:1	12	1. tofacitinib 5 mg twice daily 2. tofacitinib 10 mg twice daily 3. placebo followed by tofacitinib 5 mg††,* 4. placebo followed by tofacitinib 10 mg††	PtGA-VAS; Pain-VAS; HAQ-DI; PGJS-VAS; SF-36 PCS; SF-36 MCS; SF-36 domains; FACIT-F; EQ-5D-3L; EQ-VAS; ASQoL	395	Pfizer
OPAL Broaden 2017^19^	Multicentre	2:2:2:1:1	12	1. tofacitinib 5 mg twice daily 2. tofacitinib 10 mg twice daily 3. adalimumab 40 mg every 2 weeks 4. placebo followed by tofacitinib 5 mg††,* 5. placebo followed by tofacitinib 10 mg††	PtGA-VAS; Pain-VAS; HAQ-DI; PGJS-VAS; SF-36 PCS; SF-36 MCS; SF-36 domains; FACIT-F; EQ-5D-3L; EQ-VAS; ASQoL	422	Pfizer
SELECT-PsA1 2021^21^	Multicentre	1:1:1:1	12	1. upadacitinib 15mg once daily 2. upadacitinib 30mg once daily 3. adalimumab 40 mg every 2 weeks 4. placebo	HAQ-DI; FACIT-F; SF-36 PCS; SF-36 MCS; SAPS; PtGA; Pain; SF-36 domains; EQ-5D-5L; WPAI; itch; BASDAI 50	1705	AbbVie
SELECT-PsA2 2021^22^	Multicentre	2:2:1:1	12	1. upadacitinib 15mg once daily 2. upadacitinib 30mg once daily 3. placebo followed by upadacitinib 15†,* 4. placebo followed by upadacitinib 30†	HAQ-DI; FACIT-F; SF-36 PCS; SF-36 MCS; SAPS; PtGA; Pain; SF-36 domains; EQ-5D-5L; WPAI; itch; BASDAI 50	642	AbbVie
Mease 2023^25^	Multicentre	2:2:1:2	16	1. brepocitinib 10 mg once daily 2. brepocitinib 30 mg once daily 3. brepocitinib 60 mg once daily 4. placebo	HAQ-DI; SF-36 PCS; SF-36 MCS; PAAP; PGJS-VAS; FACIT-F	218	Pfizer

† patients switched at week 24;

†† patients switched at week 12 (3 months);

* placebo arms were analysed as one in reports at timepoint of outcome assessment;

‡ refers to the pre-defined per protocol outcomes of this study that were reported in each trial; ASQoL: Ankylosing Spondylitis Quality of Life Questionnaire; BASDAI 50: 50% improvement in Bath Ankylosing Spondylitis Disease Activity Index; EQ-5D-3L: European Quality of Life 5 Dimensions 3 Level Version; EQ-5D-5L: European Quality of Life 5 Dimensions 5 Level Version; FACIT-F: Functional Assessment of Chronic Illness Therapy – Fatigue; HAQ-DI: Health Assessment Questionnaire – Disability Index; PAAP: Patient’s Assessment of Arthritis Pain; PGJS: Patient Global Joint and Skin Assessment; PsAID9: EULAR Psoriatic Arthritis Impact of Disease; PtGA: Patient Global Assessment; SAPS: Self-Assessment of Psoriasis Symptoms; SF-36 MCS: Short Form 36 – Mental Component; SF-36 PCS: Short Form 36 – Physical Component; VAS: Visual Analogue Scale; WPAI: Work Productivity and Activity Impairment Questionnaire.

## JAKi, PROs, AND CLINICAL EVIDENCE

### PROs in a Summary

To assess the perception of disease from patients, researchers utilize a range from different instruments. Such questionaries include: the Patient’s Global assessment of Disease Activity (PtGA), which is measured with a 0–100mm visual analogue scale (VAS) and a higher score indicates increased disease activity^14^; the separate joint and skin global scores (PGJS-VAS)^14^; the Pain-VAS, which explores the level of pain^14^; the Medical Outcome Study Short Form-36 (SF-36), which assesses eight health domains through 36 questions (higher scores represent higher well-being) and the combined domain scores result in a Physical Component Summary (PCS) and a Mental Component Summary (MCS)^15^; the Functional Assessment of Chronic Illness Therapy-Fatigue (FACIT-F) which is used to investigate the level of fatigue in patients with PsA, and includes 13 questions and a total range of score of 0–52 (higher scores are associated with less fatigue)^16^; the EQ-VAS (higher score indicates better state), and the EQ-5D-3L, which measures in a 1–3 range five dimensions (higher scores indicates poorer state)^17^; the Health Assessment Questionnaire Disability Index (HAQDI), which contains 2–3 questions for 8 different sections (range 0–3, higher scores indicates greater disability) (**see Table 1**).

#### Tofacitinib

OPAL Beyond^18^ and OPAL Broaden^19^ were phase III trials that assessed the effectiveness of tofacitinib in patients with PsA, who had had an inadequate response to tumour necrosis factor inhibitors (TNFi), or conventional synthetic disease-modifying antirheumatic drugs (csDMARDs), respectively. The OPAL Beyond trial enrolled 394 patients and randomized them to tofacitinib 5 or 10 mg twice daily, and placebo. All patients were administered one background csDMARD. The OPAL Broaden trial enrolled 422 patients and randomized them to tofacitinib 5 or 10 mg twice daily, adalimumab 40 mg (once every 2 weeks), and placebo. All patients were administered one background csDMARD. Both studies had been completed by April 2016.

In both studies, both arms of tofacitinib achieved greater improvements than placebo in the PtGA-VAS and the Pain-VAS outcomes at 3 months. Furthermore, tofacitinib outperformed placebo in the PGJS score improvement from the first month of treatment. In addition, both tofacitinib interventions performed greater than placebo in the SF-36 PCS (as early as month 1) and some of its domains (physical functioning, bodily pain, vitality) at month 3. In the OPAL Beyond significant improvements were also found in the social functioning domain as well. Regarding the SF-36 MCS, interventions and placebo did not differ. Finally, tofacitinib improved greater the FACIT-Fatigue than placebo.

Another phase III randomised controlled trial (RCT)^20^ examined the effectiveness of tofacitinib in achieving PROs compared to placebo. The study was conducted between 2018 and 2021 and enrolled 131 Chinese patients with PsA. The study confirmed that tofacitinib outperforms placebo in HAQ-DI reduction as early as week 2. Additionally, tofacitinib administration was associated with greater improvements in SF-36 PCS scores versus placebo. Interestingly, that trial also reported greater improvements for the intervention on the SF-36 MCS as well compared to placebo, on the contrary with the OPAL studies.

#### Upadacitinib

Select-PsA 1^21^ and Select-PsA 2^22^ were phase III trials that assessed the effectiveness of upadacitinib in patients with PsA, who had had an inadequate response to non-biologic disease-modifying antirheumatic drugs (DMARDs), or biologic DMARDs (bDMARDs), respectively. The Select-PsA 1 trial enrolled 1705 patients and randomised them to upadacitinib 15 or 30 mg once daily, adalimumab 40 mg (once every 2 weeks), and placebo. The patients were permitted to be administered background csDMARD. The Select-PsA 2 trial enrolled 642 patients and randomized them to upadacitinib 15 or 30 mg, and placebo. The patients were permitted to be administered background csDMARD.

Both trials reported improvements in mean changes compared to placebo for both upadacitinib doses across most PROs examined. Upadacitinib outperformed placebo in PtGA, HAQ-DI, FACIT-F, and pain as early as week 2. Moreover, at week 12 upadacitinib was found to induce greater improvements in all SF-36 domains and BASDAI 50. At the same timepoint, Select-PsA 1 reported that upadacitinib was also shown to mediate greater improvements in HAQ-DI, SF-36 PCS and some of its’ domains compared to adalimumab.

#### Filgotinib

The EQUATOR study^23^ was a phase II multicentre study. In total, 131 patients were enrolled and allocated to receive filgotinib 200 mg or placebo once daily for 16 weeks. Significant improvements in the Psoriatic Arthritis Impact of Disease 9 (PsAID9) were observed in patients receiving filgotinib as early as week 4 (the mean changes from baseline [SD] at week 16 were −2.3 [1.8] and −0.8 [2.2], respectively [P < 0.0001]). Moreover, all individual PsAID9 domains were greatly improved at week 16. At weeks 4 and 16 the changes from baseline in the SF-36 PCS score were significantly higher in the filgotinib 200 mg group. Interestingly, in the SF-36 MCS score no difference between the groups was found. Finally, the authors suggest that moderate to strong negative statistically significant correlations between PsAID9 and both SF-36 PCS and MCS scores exist.

#### Deucravacitinib

A phase II double-blind RCT^24^ enrolled 203 patients with a diagnosis of PsA who had not respond to at least one prior therapy. The patients were allocated to three randomization groups: 1) oral placebo, once a day, 2) deucravacitinib 6 mg once a day, or 3) deucravacitinib 12 mg once a day for 16 weeks. Evidence of improvement at HAQ-DI scores were noticed from week 4, while mean improvements from baseline for the deucravacitinib arms were significantly greater than that of placebo at week 16 (p≤0.002). The mean change from baseline in PtGA was −13.4 for the placebo group, −28.7 and −27.6 for the 6 mg and the 12 mg group, respectively. Moreover, regarding Pain-VAS the mean change from baseline was −13.8 for the placebo group, −25.3 and −27.5 for the 6 mg and the 12 mg group, respectively. Deucravacitinib treatment at both doses was also found to improve SF-36 PCS (p≤0.0062), as well as SF-36 MCS outcomes (nominal p≤0.0263) significantly greater than placebo at week 16. Finally, the authors suggested that the deucravacitinib-mediated improvements of patient reported outcomes were independent from the dose received.

#### Brepocitinib

A phase IIb study^25^ was conducted in 11 European countries. Eligible patients were randomized to brepocitinib 60, 30, 10 mg once daily, or placebo for 16 weeks. A total of 217 participants were included in the analyses. At week 16 the change from baseline in HAQ-DI scores was significantly higher with brepocitinib 60 and 30 mg versus placebo. Furthermore, regarding pain, fatigue and SF-36 PCS greater improvements in the brepocitinib groups were found at week 16. No difference among the groups regarding SF-36 MCS was reported. Observed scores at week 16 in HAQ DI, fatigue, SF-36 PCS, and pain continued to improve up to week 52 for both brepocitinib groups.

### JAK inhibition in pain and depression: Lessons to learn from basic and translational studies

The JAK-STAT pathway occupies a pivotal position at the convergence of neurotransduction, thus facilitating the perplexed regulation of neuroinflammation and potentially its association with stress-induced pain, anxiety, depression, and established neuropsychiatric conditions. From an evolutionary perspective, JAK-STAT is imperative for maintaining homeostasis within nervous tissues, and this is evident even in phylogenetically distant species such as zebrafish.^26^ Chronic phycological stress appears to perpetuate activation of this pathway in astrocytes and microglia, leading to neuroinflammation and subsequent features of typical stress-associated neuropsychiatric disorders including synaptic disorders, and cognitive impairments.^27^ Furthermore, the downstream proinflammatory activity exerted via JAK-STAT fosters the modulation of synaptic plasticity in neurons, and interrupts the fine balance of synaptic proteins, the homeostasis of which is imbalanced in chronic pain and neuropsychiatric diseases.^28^ Its imperative role is also evident in several neurological diseases, from epilepsy and multiple sclerosis to Alzheimer’s disease.^29^

It is well known that the JAK-STATs pathway plays a role in maintaining neuropathic pain.^30^ Following nerve injury, JAK-STAT mediates astrocyte proliferation.^31^ Also, the microglial activity of the JAK-STAT3 proteins alters the functional properties of astrocytes and neurons, further indicating its contributing role in the remodelling of the spinal cord following peripheral nerve injury.^32^

JAK inhibitors exercise pleiotropic effects and may directly influence the outcome of comorbidities in patients with autoimmune rheumatic diseases such as PsA. A recent metanalysis reviewing RCTs in patients with rheumatoid arthritis concluded that treatment with JAK inhibitors have a beneficial impact on mental health of adult RA patients.^33^ The investigators found that monotherapy with JAK inhibitors improved the mental health of patients comparing that of the patients at baseline. It is noteworthy that JAK inhibitors displayed better improvement in mental health in comparison to other DMARDs or placebo. Of interest, among all the JAK inhibitors, tofacitinib showed a greater improvement in mental health followed by upadacitinib and baricitinib.^33^

The mechanism by which JAK inhibitors improves mental health remains largely elusive.^34^

The significance and likely contribution of JAK signalling pathways in the induction of depression is reinforced by the fact that JAK can regulate the expression or function of several neurotransmitter receptors; those include gamma-aminobutyric acid (GABA), cholinergic muscarinic, N-methyl-D-aspartate (NMDA) and α-amino-3-hydroxy-5-methyl-4-isoxazolpropionic acid (AMPA) receptors. All of these are directly or indirectly associated with symptoms of depression.

The fact that the selective JAK-3 inhibitor tofacitinib improves SF-36 scores the most raise the speculative expectation that depression is mostly induced by a JAK-3 phosphorylation downstream mediated axis.^33^ In support of this, there is direct evidence that stress-induced JAK3 activation is partly accomplished through the acid sphingomyelinase. In murine studies, inhibition of this enzyme diminishes Jak-3 phosphorylation and recovers hippocampal neurogenesis.^35^

A translational recent study compared the mRNA and protein expression of genes for JAK1-JAK3 and STAT1-STAT5 in patients with depressive disorders and healthy subjects and found an increased expression of JAK3 (and decreased expression of STAT1) in the group of depressed patients, reinforcing the specific role of JAK-3 in depression.^36^

Having considered the likely effect of JAK inhibitors in improving SF36 in RA and PsA, imperative issues may need to be taken into account. SF-36 assesses depression and anxiety, but standard depression and anxiety scales are essential in order to delineate the precise effect of JAK inhibitors on mental health. Of importance, several of the novel anti-depression drugs which are assessed in animal models of ‘sickness behaviour’ and in human depression clinical trials can suppress inflammation via a SAPK/MAPK and/or JAK/STAT signalling, at least *in vitro.*^37^

## DISCUSSION

JAK inhibitors have emerged as a promising treatment option for PsA, offering rapid and sustained improvement in joint and skin symptoms and overall disease activity. However, the effects of JAK inhibitors on PROs, which provide a more subjective and personal perspective on the impact of the disease and its treatment, have only recently been systematically examined.

Numerous studies have demonstrated the efficacy of JAK inhibitors in improving PROs in patients with PsA. These findings have been consistent across various patient populations, including those with active PsA, those with an inadequate response to conventional therapies, and those with comorbidities. The evidence supporting the benefits of JAKi on PROs in PsA is compelling, demonstrating consistent improvements in pain, physical function, fatigue, and quality of life.

However, a recent network meta-analysis^38^ that accessed the comparative effectiveness of available treatments for PsA on inducing PROs, executed comparisons and ranked them from best to worst. Of note the study extracted data only from the OPAL trials to account for the JAKi class of drugs. Nonetheless, the authors report that “JAKi often had the lowest efficacy”. Finally, they conclude than intravenous TNF inhibitors provide greater improvements to PROs relative to other available agents. Nevertheless, regarding some of the examined PROs, JAKi have been found to outperform treatment classes such as bDMARDs, or cDMARDs in patients with rheumatoid arthritis. In a recent meta-analysis that assessed the effect of JAK inhibitors on PROs was reported that JAK inhibitors reached better results on pain and fatigue scales compared to biologics.^39^ This study included a significant number of phase III trials with JAKi as interventions, on the contrary with that mentioned above. Whether the better performance is attributed to a more significant effectiveness of JAKi on rheumatoid arthritis, or to α low representation of the JAKi class in the PsA network meta-analysis remains to be seen.

As JAK inhibitors continue to be investigated and developed, their potential to improve patient-reported outcomes and enhance the overall well-being of individuals living with PsA remains a significant focus of research and clinical practice.
